# Extracting single-trial neural interaction using latent dynamical systems model

**DOI:** 10.1186/s13041-021-00740-7

**Published:** 2021-02-15

**Authors:** Namjung Huh, Sung-Phil Kim, Joonyeol Lee, Jeong-woo Sohn

**Affiliations:** 1 Department of Medical Science, College of Medicine, Catholic Kwandong University, Gangneung, 25601 Republic of Korea; 2 Department of Biomedical Engineering, Ulsan National Institute of Science and Technology, Ulsan, 44919 Republic of Korea; 3 Center for Neuroscience Imaging Research, Institute for Basic Science (IBS), Suwon, 16419 Republic of Korea; 4 Department of Biomedical Engineering, Sungkyunkwan University (SKKU), Suwon, 16419 Republic of Korea; 5 Translational Brain Research Center, International St. Mary’s Hospital, Catholic Kwandong University, Incheon, 22711 Republic of Korea

**Keywords:** Neural interaction, Latent dynamical systems model, Cross-correlogram, Optimized neural activity

## Abstract

In systems neuroscience, advances in simultaneous recording technology have helped reveal the population dynamics that underlie the complex neural correlates of animal behavior and cognitive processes. To investigate these correlates, neural interactions are typically abstracted from spike trains of pairs of neurons accumulated over the course of many trials. However, the resultant averaged values do not lead to understanding of neural computation in which the responses of populations are highly variable even under identical external conditions. Accordingly, neural interactions within the population also show strong fluctuations. In the present study, we introduce an analysis method reflecting the temporal variation of neural interactions, in which cross-correlograms on rate estimates are applied via a latent dynamical systems model. Using this method, we were able to predict time-varying neural interactions within a single trial. In addition, the pairwise connections estimated in our analysis increased along behavioral epochs among neurons categorized within similar functional groups. Thus, our analysis method revealed that neurons in the same groups communicate more as the population gets involved in the assigned task. We also showed that the characteristics of neural interaction from our model differ from the results of a typical model employing cross-correlation coefficients. This suggests that our model can extract nonoverlapping information about network topology, unlike the typical model.

## Introduction

Information communication via spike trains of neurons in populations is a core computational process that enables many brain areas to execute their roles, which include encoding of stimuli, decision-making, and high-level cognition [1]. To understand these processes, therefore, the effects of spike trains across neuronal populations must be determined according to their specific network structures [2–6]. For this purpose, a variety of network theoretical models have been developed for the analysis of neuronal population dynamics and the description of network topology, e.g., modules, hubs, and rich-clubs [7–9]. From analyses of pairwise correlations, a recent study showed that the functional single neuron network of the macaque monkey frontoparietal area during active behavior has a highly complex topology that includes small-worldness, hubs, and rich-clubs consisting of oscillatory neurons synchronized in specific frequency bands [9]. To infer neural network topology, many studies have focused on quantifying correlations in the number of spikes or in the spike times of pairwise neurons. One conventionally used quantification method is the cross-correlogram (CCG) coefficient, which measures the co-occurrence of spikes of the pairs within a given time bin [10]. To assess temporal correlations rather than the degree of synchronous firing, alternative quantification methods can be applied, such as the correlation index [11] or spike-time tiling coefficient [12], which are derived from the temporal order of pairwise spike trains. Given sufficient data, it is also possible to make use of nonlinear methods such as mutual information [13] and transfer entropy [14] in information theory.

Measuring sets of neural responses across trials is common to estimate the firing rates of spike trains because spikes are highly variable and noisy at the single-trial level. The averaged values across trials, however, obfuscate information of population dynamics at certain times in behavioral epochs or in the trials. Since estimating neural activities in single trials is challenging because of variability and deficient sampling, several studies have exploited the dimensionality reduction method adapted in the latent variable space rather than the neuronal space to measure population activities at the single-trial level [15–29]. Models with independent factors underlying population activities can extract neural population states in single trials [15, 16]. Recent studies have reported analysis of single-trial population activities using linear dynamical models across time [17–20] or nonlinear dynamical models allowing switches of states at given times [21, 22]. Event-dependent dynamical systems models [22] are variant models compromising linear and nonlinear methods that allow switches at the cued times between adjacent epochs. In addition, artificial recurrent neural networks infer single-trial neural population dynamics based on the assumption that the networks can generate neural data using a machine-learning method [23]. In nonhuman primates, population activities in diverse brain regions are low dimensional in many instances [19, 30–37]; therefore, it can be assumed that population activity in single trials can be estimated via a latent dynamical systems model with much lower dimensions than the population size.

Estimations of population activity are useful for elucidating the slow-varying neural dynamics governed by shared inputs in a single trial; however, the recovery of transient changes evoked by other neurons’ spikes is limited because the temporal precision of the latent model is generally insufficient for capturing these interactions. The intrinsic timescale of spike effects is about tens of ms and below 200 ms at most [38], whereas neural populations sustain their activity in the same-shared activity space within an epoch that generally lasts longer than hundreds of ms [22]. To address this issue, it is possible to calculate CCGs of pairs of neurons by two different ways that CCGs are calculated using spike trains or rate estimates. In typical fashion, CCGs are calculated using spike trains, but they include the effects caused by shared motives such as common inputs or synchronization across the populations as well as the direct effects of the other neurons in the population. To quantify these shared terms, the CCGs can be calculated using estimates of population activity inferred by a latent dynamical systems model. The transient effects between pairs of neurons can be surmised by comparing two CCGs according to spike trains or estimates of populate activity. Estimates of population activity can then be revised in light of transient effects, and the degree to which both transient and shared effects contribute to the revised estimate can be evaluated.

In the present study, we applied the above-described method to a dataset from an open database in which data were simultaneously recorded from the anterior lateral motor (ALM) cortex of mice while they were executing a two-alternative-forced choice task [29, 39–43]. We found that neural interactions varied significantly over time in a trial and that neurons could be classified based on the degree of other neurons’ effects in each behavioral epoch. We also showed that network structures were dependent on the task-related epochs, implying that interactions between neurons were not stationary but instead adjusted to the relative importance of the epochs. Compared with a model containing correlation coefficients, which are widely used to evaluate functional connectivity, our model could improve judgment of the underlying organization of transient neural activities.

## Materials and methods

### Application to mice ALM cortex neurons

**Fig. 1:**
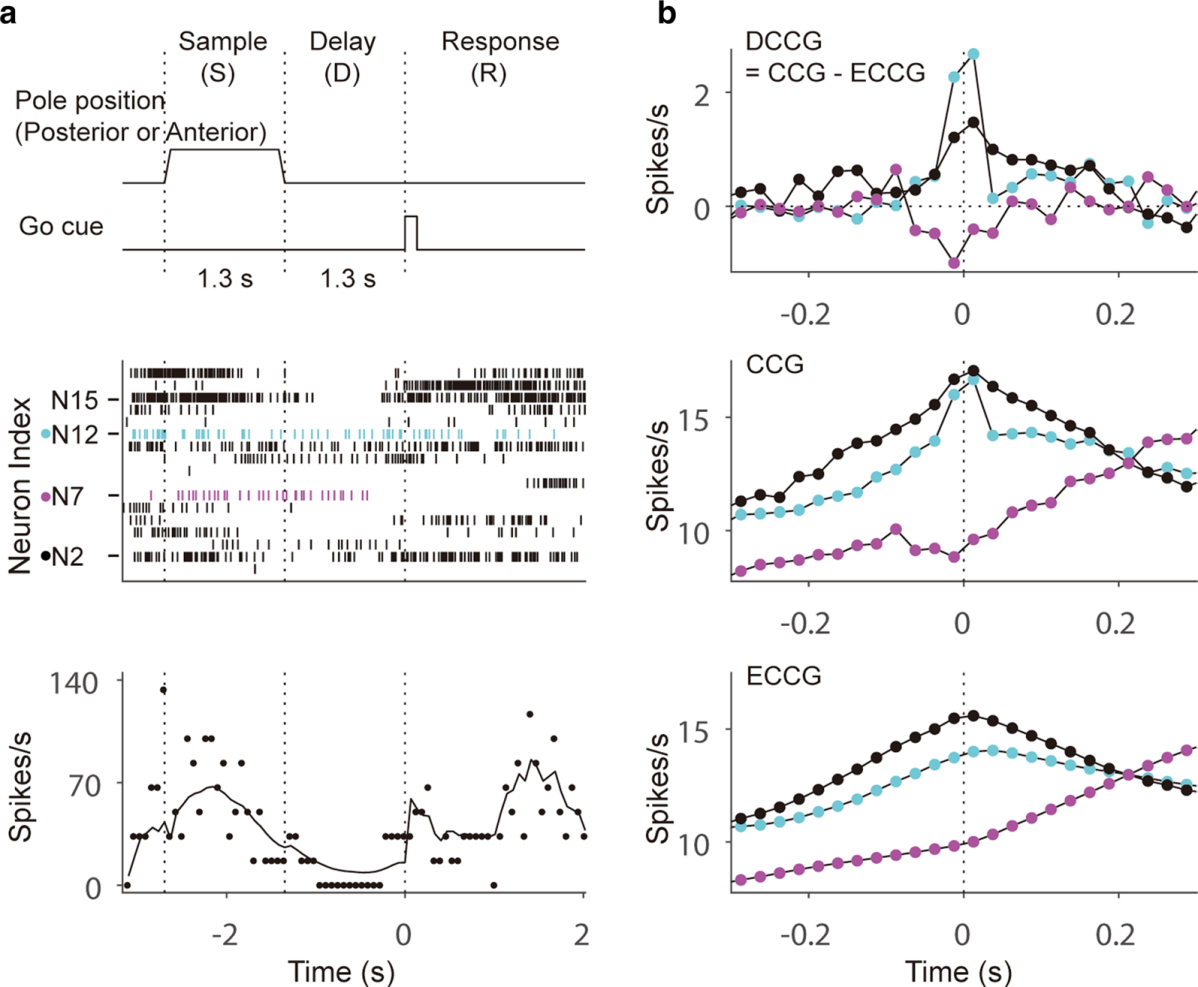
Schematics of the behavioral task and examples of neural activities and cross-correlograms. **a** In a sample epoch, the pole is presented at one location (posterior or anterior) and mice are trained to lick a corresponding port to report pole location in a response epoch, which was signaled by an auditory cue after a 1.3-s delay epoch (upper panel). Examples of spike trains in a single trial (middle panel); the number of spikes in time bins and the rate estimate of neuron N15 (lower panel). **b** An example of the differences among cross-correlograms (upper panel), spike trains (middle panel), and rate estimates (lower panel). Neural activities of the target neuron (N15) are aligned based on the different reference neurons (N2: black; N7: magenta; N12: cyan)

We applied the above-described analysis method to a dataset from alternative choice tasks conducted in previous studies [29, 39–43]. In this research, trained mice reported pole position (posterior or anterior) by licking one of two targets (left in an ipsi trial and right in a contra trial) following a delay epoch after pole presentation. After a 1.3-s delay, mice responded to an auditory signal indicating the onset of the response epoch (Fig. 1a, upper panel). Extracellular spikes were recorded on the left-hemisphere ALM cortex using 32-channel NeuroNexus silicon probes (19 mice) or 64-channel Janelia silicon probes (20 mice). The extracellular recording traces were bandpass-filtered (300–6000 Hz) and then sorted using JRclust if events exceeded an amplitude threshold. Based on spike width, cells were classified as pyramidal cells (width > 0.5 ms) or fast-spiking interneurons (width < 0.35 ms). For a more detailed explanation of the electrophysiological recording method, consult the studies cited earlier [40, 42, 43]. To assess the effects of ensemble inputs from other neurons, we analyzed only the sessions that included > 15 neurons (9 of 40 sessions).

### Estimating the dynamics of ALM cortex neural activities

Neural dynamics are highly complicated and stochastic, resulting in diverse responses even in comparable cases. To resolve these difficulties, the extraction of shared neural activity (SNA) in a low-dimensional latent space has been developed to determine the dynamics of population activity as an alternative to directly assessing raw neural activities. For this purpose, we used a previously introduced model [22], i.e., an event-dependent linear dynamical systems (EDLDS) model that allows switches of behavioral epochs as follows:

1 $$r\left(t\right) = V\left(s\right)x\left(t\right) + {{r}_{0 }+ V}_{0}(t)$$

2 $$dx\left(t\right) / dt = W\left(s\right)x\left(t\right) + {W}_{0}(t)$$

The latent variable $x\left(t\right)$ is an ${N}^{L}$-dimensional vector (${N}^{L}$: the dimension of the latent space) updated by matrix $W\left(s\right)$ in the s-epoch (s: sample, delay, response). The term ${W}_{0}(t)$ represents random fluctuation independent of the term updated by matrix $W\left(s\right)$ in the latent space. The rate estimate $r(t)$ is an ${N}^{P}$-dimensional vector (${N}^{P}$: the number of neurons in the population) calculated by the projection matrix $V\left(s\right)$ in the s-epoch. The term ${V}_{0}(t)$ denotes the residual that is not explained by the latent variables.

*Temporal interaction estimated with CCGs*. Since the EDLDS model captures slow-varying dynamics of the neural response, neural components that reflect temporal fluctuations must be introduced into the model. One such possible component is the interaction between neurons over a finite time scale. We assumed that a neuron was affected by all the other neurons in the population and that the rate estimate calculated in Eq. 1 could be revised by the integration of these interactions. Accordingly, we calculated CCGs between all pairs of neurons from their spike trains ${C}_{ij}(k\tau )$ as follows:

3 $${C}_{ij}(k\tau )=\frac{1}{M}{\sum }_{m=1}^{M}\frac{1}{{N}_{j}^{m}}{\sum }_{l=1}^{{N}_{j}^{m}}{n}_{i}^{m}\left({t}_{jl}^{m},k\right)/\tau$$

where $j$ ($i$) is the index of a reference (a target) neuron affecting (affected by) the other neuron. $M$ and ${N}_{j}^{m}$ are the number of trials and the number of spikes of the reference neuron during trial *m*, respectively, and ${n}_{i}^{m}\left({t}_{jl}^{m},k\right)$ denotes the number of spikes of the target neuron included in the *k*-th time lag range ${t}_{jl}^{m} + \left(k - 1\right)\tau \le t < {t}_{jl}^{m} + k\tau$ after the *l*-th spike time ${t}_{jl}^{m}$ of the reference neuron. We found similar results with various temporal precisions from $\tau =15 ms$ to $\tau =45 ms$ for the transient effects; thus, we heuristically selected the temporal precision $\tau =25 ms$ because the results of analysis using this value showed greater distinction in neural interactions according to the epoch. In a similar manner, the estimated CCG (ECCG) from the SNA of a latent model ${C}_{ij}^{E}(k\tau )$ can be represented as:

4 $${C}_{ij}^{E}(k\tau ) = \frac{1}{M}{\sum }_{m = 1}^{M} \frac{1}{{N}_{j}^{m}}{\sum }_{l = 1}^{{N}_{j}^{m}}\langle {r}_{i}^{m}\left({t}_{jl}^{m},k\right)\rangle$$

where $\langle {r}_{i}^{m}\left({t}_{jl}^{m},k\right)\rangle$ denotes the mean rate estimate of the target neuron in the *k*-th time lag range ${t}_{jl}^{m }+ \left(k - 1\right)\tau \le t < {t}_{jl}^{m} + k\tau$ after the *l*-th spike time ${t}_{jl}^{m}$ of the reference neuron. In line with ${n}_{i}^{m}\left({t}_{jl}^{m},k\right)/\tau$ in Eq. 3, which denotes the rate in the range ${t}_{jl}^{m} + \left(k - 1\right)\tau \le t <{t}_{jl}^{m} + k\tau$ derived from the spike trains of the *i*-th neuron, the counterpart $\langle {r}_{i}^{m}\left({t}_{jl}^{m},k\right)\rangle$ in the ECCG (Eq. 4) is the rate in the same range derived from the rate estimate of the *i*-th neuron (Eq. 1). The difference between the CCG and ECCG (i.e., the DCCG) $\Delta {C}_{ij}\left(k\tau \right)$ can then be represented as follows:

5 $$\Delta {C}_{ij}\left(k\tau \right) ={ C}_{ij}\left(k\tau \right) - {C}_{ij}^{E}\left(k\tau \right)$$

### Optimization of neural activities

SNA effectively describes the slow-varying population activity governed by the shared inputs. However, recovering the transient changes evoked by the spikes of the connected neurons is difficult due to the short time variability of the interaction effect compared with the temporal precision of the latent model. For increasingly accurate estimates that include these transient effects over a relatively short time scale, the rate estimate in the given time bin of the latent model can be corrected using the CCG and ECCG. Since the interaction between neurons might change across times or trials, we allowed the CCG and ECCG to depend on both variables. We calculated the CCG and ECCG from the spike trains in the neighboring 20 bins of 20 trials (we obtained similar results using different values, e.g., 15 or 30 bins or trials; data not shown). For simplicity, we used only one-time lag before and after the spike of the reference neuron (${C}_{ij}\left(\tau \right), {C}_{ij}\left(-\tau \right)$) and then the corrected rate estimate ${r}_{i,C}^{T}(t)$ as follows:

6 $${r}_{i,C}^{T}(t) = \sum_{j \ne i}\left[{\delta }^{+ }\left(\left\{{t}_{j}\right\},t\right)\left(\frac{{C}_{ij}\left(\tau \right)}{{C}_{ij}^{E}\left(\tau \right)}\right)\left(\frac{{C}_{ij}^{E}\left(\tau \right)}{\sum_{j\ne i}{C}_{ij}^{E}\left(\tau \right)}\right){r}_{i}^{T} + {\delta }^{-}\left(\left\{{t}_{j}\right\},t\right)\left(\frac{{C}_{ij}\left(-\tau \right)}{{C}_{ij}^{E}\left(-\tau \right)}\right)\left(\frac{{C}_{ij}^{E}\left(-\tau \right)}{\sum_{j\ne i}{C}_{ij}^{E}\left(-\tau \right)}\right){r}_{i}^{T}\right]$$

where ${\delta }^{+}\left(\left\{{t}_{j}\right\},t\right) = 1$ or 0 (${\delta }^{-}\left(\left\{{t}_{j}\right\},t\right) = 1$ or 0) if neuron *j* fires (or does not fire) in the time lag range $\left[\mathrm{t},t + \tau \right]$ ($\left[t - \tau ,t\right]$), *T* denotes the time bin index of the latent model, and $0 \le t <{ S}_{bin}$ (${S}_{bin}$: time bin size of the latent model). We used ${S}_{bin}$= 67.4 ms following a previous study [22]. The rate estimate ${r}_{i}^{T}$ represents the SNA of the *i-th* neuron in the given time bin $T$. Because of the effects of the other neurons’ spikes, the mean of the corrected rate estimate ${r}_{i,C}^{T}(t)$ in the given time bin *T* becomes different from the rate estimate ${r}_{i}^{T}$. To resolve this disagreement, we normalized the corrected rate estimate to match with the SNA; the transient SNA (TSNA) ${r}_{i,TSNA}^{T}\left(t\right)$ was then represented as follows:

7 $${r}_{i,TSNA}^{T}\left(t\right)= \frac{{r}_{i}^{T}}{\langle {r}_{i,C}^{T}(t)\rangle }\sum_{j \ne i}\left[{\delta }^{+}\left(\left\{{t}_{j}\right\},t\right)\left(\frac{{C}_{ij}\left(\tau \right)}{\sum_{j\ne i}{C}_{ij}^{E}\left(\tau \right)}\right){r}_{i}^{T} +{ \delta }^{-}\left(\left\{{t}_{j}\right\},t\right)\left(\frac{{C}_{ij}\left(-\tau \right)}{\sum_{j\ne i}{C}_{ij}^{E}\left(-\tau \right)}\right){r}_{i}^{T}\right]$$

where $\langle {r}_{i,C}^{T}(t)\rangle$ is the mean of ${r}_{i,C}^{T}(t)$ in the given time bin $T$. Since the contributions of transient effects to the neural response vary depending on the time bin, we found the optimal degree of SNA and TSNA contributions by maximizing the likelihood $L\left(\left\{{t}_{i}\right\}\right)$ of the target neuron’s spike times in a given time bin as follows:

8 $$L\left(\left\{{t}_{i}\right\}\right) = \sum_{t = 1}^{{S}_{bin}}\left({\delta }_{t,\left\{{t}_{i}\right\}}P\left(t\right) + (1 -{ \delta }_{t,\left\{{t}_{i}\right\}})(1 - P(t)\right)$$

where $\left\{{t}_{i}\right\}$ denotes the spike trains of neuron *i* and ${\delta }_{t,\left\{{t}_{i}\right\}} = 1$ or 0 if $t\in \left\{{t}_{i}\right\}$ or does not, respectively. The probability $P(t)$ of firing at time *t* is calculated using the soft-max selection rule $P(t) = \left(\alpha {r}_{i,TSNA}^{T}\left(t\right) + (1 - \alpha ) {r}_{i}^{T}(t)\right)\delta t$, where $\alpha = 1 / (1 +{ e}^{-Q})$. We set a discrete time step of $\delta t={S}_{bin}/60$(${S}_{bin}$= 67.4 ms: time bin size of the latent model) for all calculations in this work. The parameter $\alpha$ denotes the degree to which TSNA contributes and it is determined by *Q*. The optimized neural activity (ONA)${r}_{i,ONA}^{T}\left(t\right)$ is then represented as follows:

9 $${r}_{i,ONA}^{T}\left(t\right) = { \alpha }_{*}{r}_{i,TSNA}^{T}\left(t\right) + (1 - {\alpha }_{*}) {r}_{i}^{T}$$

where ${\alpha }_{*}$ denotes the value maximizing $L\left(\left\{{t}_{i}\right\}\right)$, i.e., ${\alpha }_{*} = 1 / (1 + {e}^{-{Q}_{*}})$ and ${Q}_{*} = arg{max}_{Q} L\left(\left\{{t}_{i}\right\}\right)$.

### Neural interactions estimated by Kullback–Leibler divergence

To assess how SNA varies by the transient effects of other neurons, we compared the probability distributions of the spike trains, i.e., Kullback–Leibler divergences, between the ONA and SNA (i.e., the DONA) ${D}_{T}^{KL}({P}_{T}^{ONA}\left(t\right)|{P}_{T}^{SNA})$ as follows:

10 $${D}_{T}^{KL}\left({P}_{T}^{ONA}\left(t\right)|{P}_{T}^{SNA}\right)= \sum_{t = 1}^{{S}_{bin}}{P}_{T}^{ONA}\left(t\right)log\left(\frac{{P}_{T}^{ONA}\left(t\right)}{{P}_{T}^{SNA}}\right)$$

where ${P}_{T}^{SNA}$ (${P}_{T}^{ONA}\left(t\right)$) denotes the probability of firing for SNA(ONA) at time *t* in the given time bin $T$. To identify interactions induced by a single reference neuron’s spike train but not by all other neurons (as with DONA), we defined the pairwise Kullback–Leibler divergence between ONA and SNA (i.e., the PDONA) ${D}_{T}^{KL}\left({P}^{ONA}\left({s}_{n}\right)|{P}^{SNA}\left({s}_{n}\right)\right)$ as follows:

11 $${D}_{T}^{KL}\left({P}^{ONA}\left({s}_{n}\right)|{P}^{SNA}\left({s}_{n}\right)\right) = \sum_{{s}_{n}\in S(n)}{P}^{ONA}\left({s}_{n}\right)log\left(\frac{{P}^{ONA}\left({s}_{n}\right)}{{P}^{SNA}\left({s}_{n}\right)}\right)$$

12 $$P\left({s}_{n}\right) = \frac{\prod_{k = 1}^{n}\left({\delta }_{1,{s}_{n}\left(k\right)}{r}_{i}\left({t}_{j}^{k}\right)\delta t+ (1 - {\delta }_{1,{s}_{n}\left(k\right)})(1 - {r}_{i}({t}_{j}^{k})\delta t)\right)}{\sum_{{s}_{n}\in S(n)}\prod_{k = 1}^{n}\left({\delta }_{1,{s}_{n}\left(k\right)}{r}_{i}\left({t}_{j}^{k}\right)\delta t +(1 -{ \delta }_{1,{s}_{n}\left(k\right)})(1 -{ r}_{i}({t}_{j}^{k})\delta t)\right)}$$

where ${s}_{n}$ is an *n*-dimensional vector with 0 or 1 denoting spike existence or nonexistence of the target neuron *i* in the time step $\delta t$ after *n*-consecutive spike times of the reference neuron *j* ($S(n)$: the entire space including all the possible cases of ${s}_{n}$) closest to the time bin *T*, whereas ${t}_{j}^{k}$ denotes the *k*-th spike time of the reference neuron *j*, and ${\delta }_{1,{s}_{n}\left(k\right)}=$ 1 or 0 if the *k*-th element of ${s}_{n}$ (${s}_{n}(k)$) is 1 or 0, respectively. $P\left({s}_{n}\right)$ represents the probability of the series of the target neuron firing or not firing after the firing of the reference neuron. Hence, PDONA measures how each of the reference neuron’s *n*-consecutive spikes modify the probability of the target neuron firing after the moment at which the corresponding reference neuron spikes. We tested various cases of *n* spikes of reference neurons (from *n* = 5 to *n* = 8); we chose to use *n* = 6 and excluded cases in which the number of spikes by the reference neuron was < 10 in the trial (we obtained similar results for *n* = 5, 7, or 8; data not shown).

### Logistic regression analysis for decoding lick responses

We evaluated how the neural interaction improved performance in decoding lick responses through logistic regression analysis, including spike counts and DONA (full model), as follows:

13 $$log\left({P}_{ipsi }/ (1 - {P}_{ipsi})\right) ={ c}_{0} +{ c}_{sp}\mathrm{log}\left({N}_{sp}\right)+{ c}_{DONA} \mathit{log}\left(DONA\right)$$

where the log-odds for the probability of the lick of the left target in the ipsi trial (${P}_{ipsi}$) were fitted using the log of the spike counts ($\mathrm{log}({N}_{sp})$) and the log of DONA ($\mathit{log}\left(DONA\right)$). By comparing Akaike’s information criterion (AIC) between the full model and reduced model including only the spike counts, we determined the optimal model for decoding lick responses.

### Cross-correlation coefficients

To compare DONA with a typical measure of neural interaction, we calculated the cross-correlation coefficients of neuron pairs in the given time bin. For direct comparison with DONA, we defined a normalized correlation coefficient (NCC) ${\rho }_{i}$, which assumes the mean correlation between a single neuron *i* and all the other neurons as follows:

14 $${\rho }_{i} = \sqrt{\sum_{j \ne i}\frac{{\rho }_{ij}^{2}}{{N}^{P} - 1}}$$

where ${N}^{P}$ is the number of neurons in the population and ${\rho }_{ij}$ is the cross-correlation coefficient between neuron *i* and neuron *j*.

## Results

### Population activities and CCGs

To demonstrate the neural interactions in an ALM cortex, we analyzed neural data collected from mice as they executed a delayed response task (Fig. 1a, upper panel). ALM neurons showed complex and variable activities, as shown in the example raster plot and firing rates from a single trial (Fig. 1a, middle and lower panels). For exemplification of different CCGs from the spike trains and rate estimates, we provide CCGs for three different pairs of neurons. N15 is the target neuron affected by the other neurons, i.e., the reference neurons; we show the effects of only three reference neurons, N2, N7, and N12, on the target neuron for the purpose of improving visibility. Examples of CCGs, ECCGs, and DCCGs for three reference–target pairs are shown in Fig. 1b. Both the CCGs and ECCGs were sustained over hundreds of ms as they moved farther away from the reference neuron’s spike time (time = 0). DCCGs, however, approached zero after a few bins (about tens of ms), implying that the DCCG captures the transient effect near time zero by offsetting the shared effects of both the CCG and ECCG remaining in the longer time range.

### Localized neural interaction terms at specific behavioral epochs

**Fig. 2:**
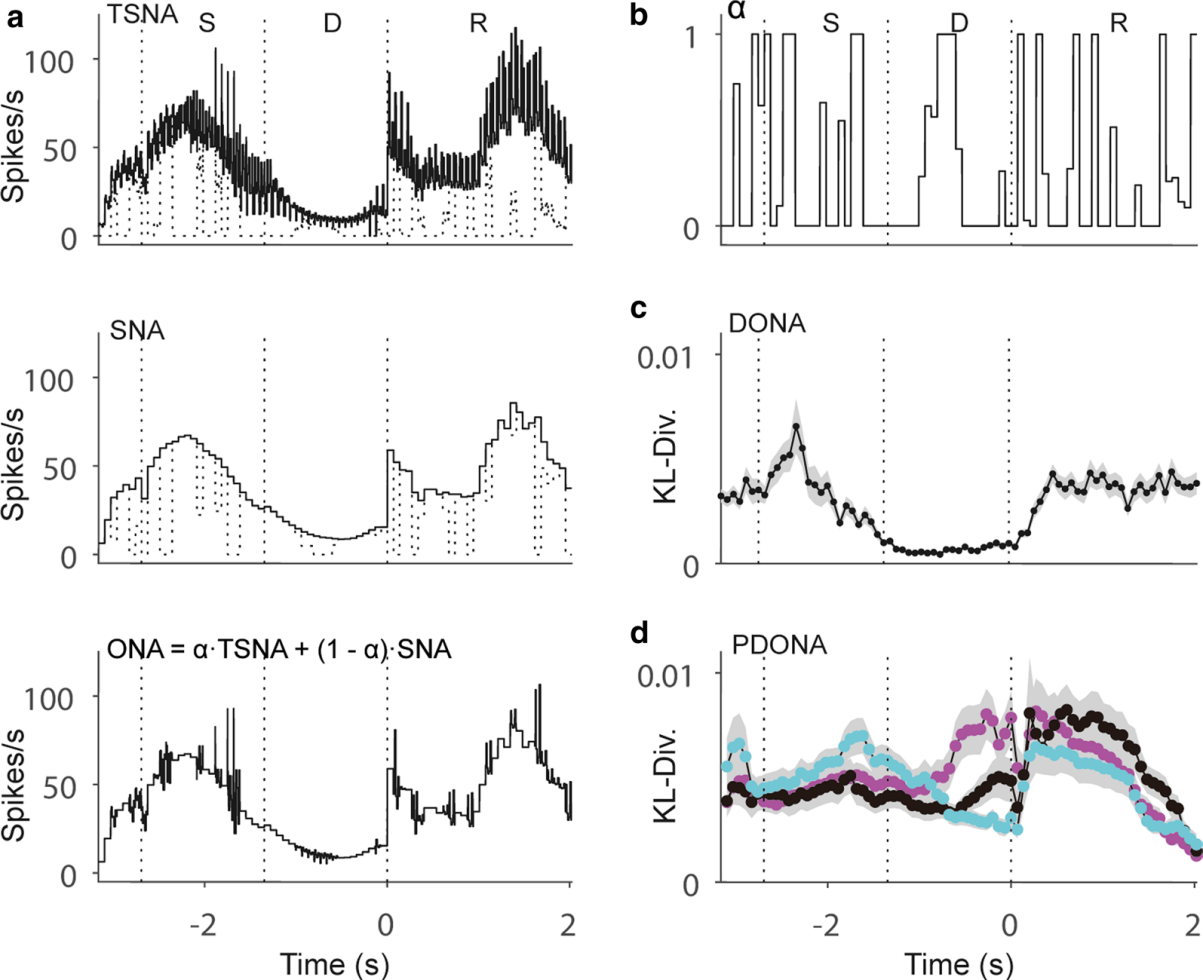
Examples of rate estimate revision and Kullback–Leibler divergence. **a**, **b** For neuron N15 shown in Fig. 1, optimized neural activity (ONA; lower panel) was calculated using transient SNA (TSNA; solid line in upper panel), shared neural activity (SNA; magenta line in middle panel), and the parameter $\alpha$ that regularized their contributions. The dotted lines in the upper and middle panels represent the TSNA and SNA included in ONA, as determined by $\alpha$. **c**, **d** Examples of Kullback–Leibler divergence of a single neuron (DONA) for neuron N15; pairwise Kullback–Leibler divergence of single pairs of neurons (PDONA) of the reference neurons N2 (black), N7 (magenta), and N12 (cyan) to the target neuron N15. The vertical lines demark the behavioral epochs, i.e., S: sensory epoch; D: delay epoch; and R: response epoch. Examples are the averages of all the trials in the given session. Shaded regions denote the standard error of the mean (SEM)

**Fig. 3:**
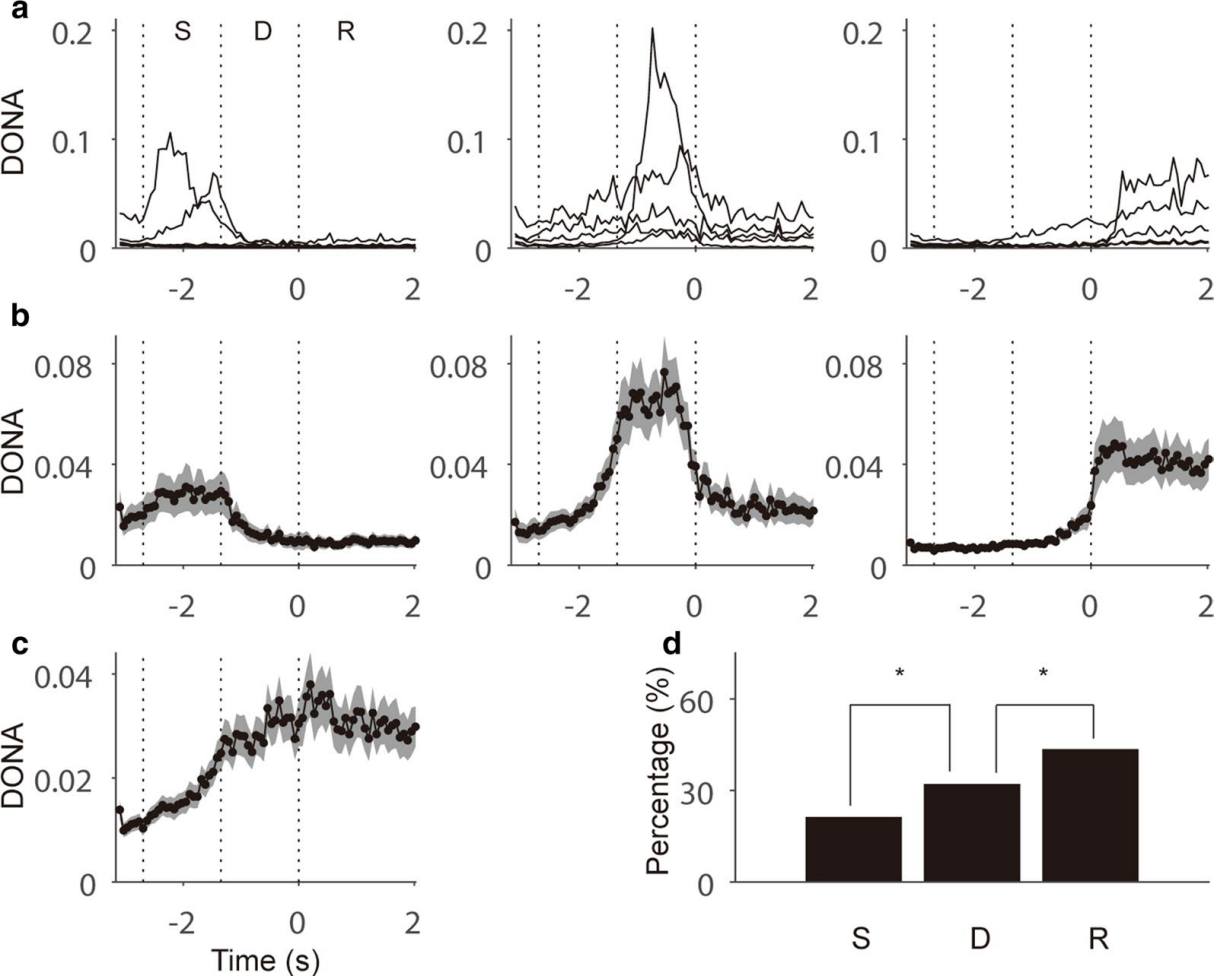
Epoch-dependent neural interactions and proportions of neurons with larger neural interactions. **a** Example DONAs of neurons showing significantly larger values of DONA in the sample (left), the delay (middle), and the response (right) epochs (S: 6; D: 5; R: 5) for 22 neurons from session 37. **b** Mean DONAs for all the sessions are sorted in the same manner as in (**a**) and the shaded region represents the standard error of the mean (SEM). **c** Mean DONAs for all the neurons regardless of significance in epochs. **d** Percentage of neurons with significantly larger DONA values in each epoch (*p < 0.05)

In our analysis, DONA is a quantity with which we can judge how the probability distribution of the ONA differs from that of the SNA, which is acquired from the latent model (Eqs. 1–2). Figure 2a provides an example of the optimization of the activities of the target neuron N15 (Fig. 1b), including all the effects of the other (reference) neurons, with parameter $\alpha$ deciding the degree of SNA and TSNA contributions (Fig. 2b). Since ONA is the optimal neural activity between the transient component from other neurons and shared components due to behavior, larger values of DONA can be interpreted as more interactions occurring with the other neurons. Neuron N15 in Fig. 2c, for example, shows more interactions with other neurons during the sensory (indicated by S) and response (indicated by R) epochs compared with the interaction observed during the delay epoch (indicated by D). For pairwise interactions, we showed PDONAs of three pairs, which represent the effects of three reference neurons N2, N7, and N12 on the target neuron N15 shown in Fig. 1b (Fig. 2d). It should be noted that the DONA of the target neuron N15 in Fig. 2c was the interaction integrated with all other neurons, although we only showed the results of the three reference neurons to improve visibility. To characterize the neural interactions of all neurons, we classified them in conformity with the epochs in which their DONAs were maximized. First, to quantify the effect over time in a trial, we averaged DONA across trials. Neurons were considered to show more interactions with other neurons in an epoch if the magnitudes of DONA in the epoch (20 bins per epoch) were larger than the DONA magnitudes in the other two epochs (Wilcoxon rank sum test, p < 0.05). In an example session shown in Fig. 3a (session no. 37), DONAs were significantly larger; therefore, they were well localized in specific epochs. Among 22 neurons, 6 (27%), 5 (23%), and 5 (23%) showed more interactions with other neurons in the sample, delay and response behavioral epochs, respectively. For the neurons of all sessions (157 neurons in 9 sessions), most (111 of 157; 70.70%) showed maximum values of DONA consistently in single epochs (Fig. 3b). This result implies that the effects of neural interactions on single neurons are rather concentrated on a moment of information processing than on being maintained throughout the entire trial. The remaining neurons showed larger values of DONA during the two epochs (26 of 157; 16.54%) or no larger values (20 of 157; 12.74%).

### Tendency of increases of neural interactions toward the end of a trial

To determine the overall variation of DONA during the progress of epochs, we simply averaged DONA values for all the neurons regardless of their statistical significance in epochs (Fig. 3c). The mean DONA value increased and reached a peak soon after the response onset. Significantly more neurons showed larger DONA values in the latter epochs, in agreement with the results of mean DONA values (p < 0.05, *χ*^2^ test; Fig. 3d). The number of neurons with larger DONA values was 33, 50, and 68 of 157 total neurons during the sample, delay, and response epochs, respectively; thus, there were significant differences between the behavioral epochs (p < 0.05 between S and D and between D and R, both *χ*^2^ tests).

**Fig. 4:**
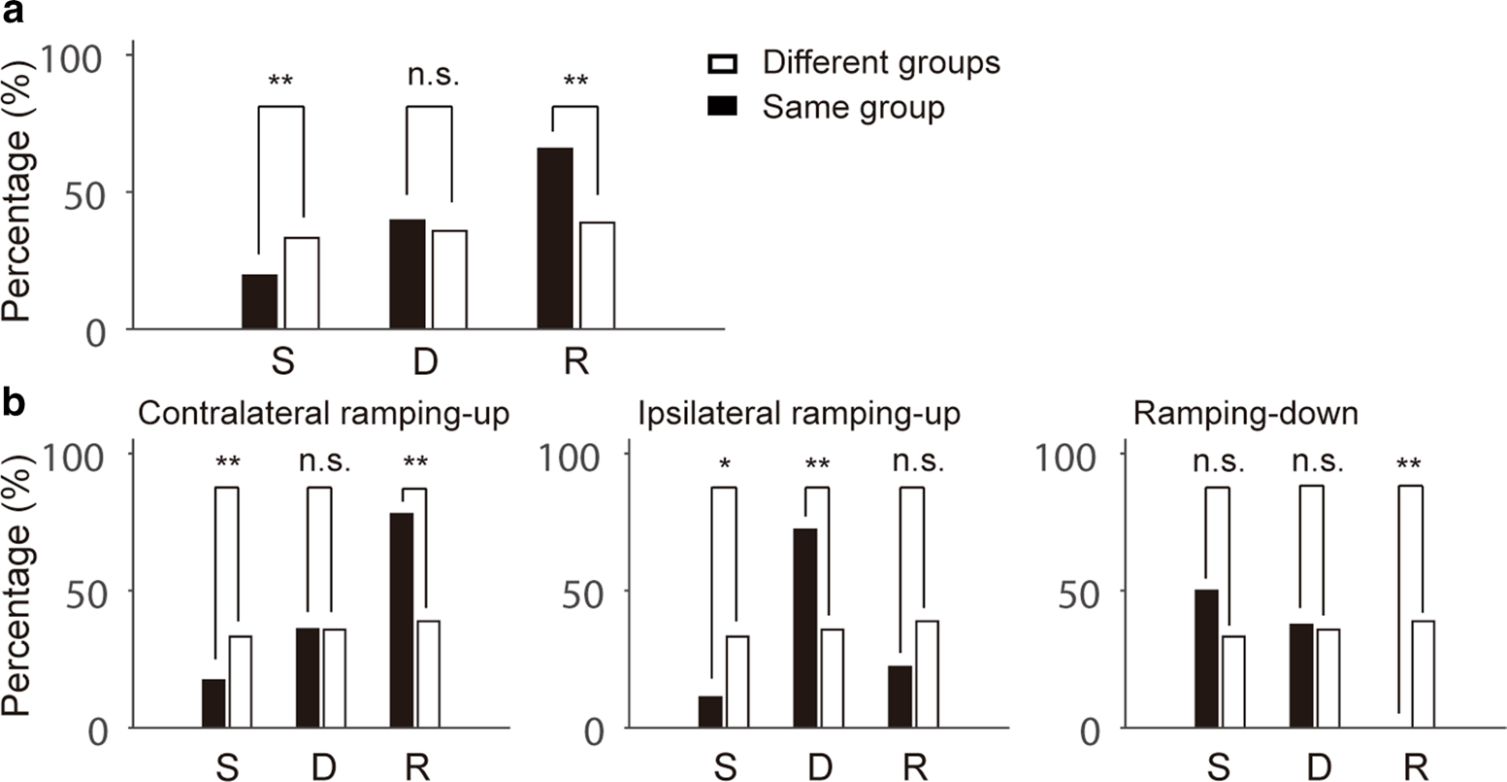
Analysis of PDONA based on defined groups of neurons. **a** Percentage of pairs of neurons for which PDONAs had significantly larger values in an epoch. Filled and empty bars represent pairs of neurons that belong to the same and different groups, respectively. **b** Percentage of pairs of neurons calculated in the same manner as in (**a**), but for pairs of neurons in contralateral ramping-up (left panel), ipsilateral ramping-up (middle panel), and ramping-down (right panel) groups (*p < 0.05; **p < 0.001; *n.s*.: not significant)

In the dataset used for this analysis, Hidehiko et al. [43] categorized neurons in the ALM cortex into four functional groups, namely contralateral ramping-up, ipsilateral ramping-up, ramping-down, and the rest based on the selectivity of the lick responses and the neurons’ ramping activities. We investigated whether the neuronal interaction term DONA was related to these functional neuron groups. According to the categorization of Hidehiko et al. [43], we placed 52, 17, 19, and 69 neurons into the contralateral ramping-up, ipsilateral ramping-up, ramping-down, and remaining groups, respectively. Secondly, we calculated PDONAs between neurons belonging to the same functional groups and the different functional groups separately. As was the case for DONA, pairs of neurons were considered to show larger interactions in an epoch if the magnitudes of PDONA in the epoch (20 bins per epoch) were larger than the magnitudes in the other two epochs (Wilcoxon rank sum test, p < 0.05). The proportion of pairs with larger values of PDONA in each epoch is shown Fig. 4a (filled and empty bars show pairs of neurons in the same functional group and different groups, respectively). Significantly more pairs in the same functional group showed larger PDONA values in the latter epochs in accordance with the DONA results shown in Fig. 3d (Fig. 4a, black bars). In the same functional group, the pairs of neurons showing larger PDONA values were 36, 73, and 121 of 184 total pairs during the sample, delay, and response epochs, respectively (p < 0.01 between S and D and between D and R, both *χ*^2^ tests). However, for pairs of neurons belonging to different functional groups, there was no significant difference in the number of pairs with larger PDONA values between epochs (Fig. 4a, empty bars): 374, 403, and 437 of 1,125 total pairs during the sample, delay, and response epochs, respectively (p = 0.199 between S and D; p = 0.138 between D and R; both *χ*^2^ tests). These results imply that the increase in neural interactions in the latter epoch was due to increased functional connections between neurons in the same functional group.

The number of pairs of larger PDONA values was significantly different in the sample and response epochs but not in the delay epoch (delay: p = 0.314, *χ*^2^ test). In the sample epoch, the proportion of pairs with larger PDONA values was significantly higher when neurons in the pairs belonged to different functional groups (p < 0.01, *χ*^2^ test). However, the opposite was true in the response epoch in which the proportion of pairs of neurons in the same functional group was higher (p < 0.01, *χ*^2^ test). We also reanalyzed the data for each group separately (Fig. 4b). The numbers of pairs of neurons in the contralateral ramping-up, ipsilateral ramping-up, and ramping-down group were 150, 18, and 16 of 184 total pairs of neurons. It should be noted that we did not analyze the fourth group (the rest) separately because neurons in the fourth group were just the remainders that could not be classified into any of the three groups discussed above. Although the results for the contralateral ramping-up group were identical to those of all the groups shown in Fig. 4a (Fig. 4b, left panel), the PDONAs of the other groups had different distributions when compared with the overall tendency to increase along the epoch (Fig. 4b, middle and right panels). In the contralateral ramping-up group, the pairs of neurons showing larger PDONA values were 26, 54, 117 of 150 total pairs during the sample, delay, and response epochs, respectively (p < 0.01 between S and D and between D and R, both *χ*^2^ tests). In the ipsilateral ramping-up group, the pairs of neurons showing larger PDONA values were 2, 13, 4 of 18 total pairs during the sample, delay, and response epochs, respectively (p < 0.01 between S and D and between D and R, both *χ*^2^ tests). In the ramping-down group, the pairs of neurons showing larger PDONA values were 8, 6, 0 of 16 total pairs during the sample, delay, and response epochs, respectively (p = 0.48 between S and D; p < 0.01 between D and R; both *χ*^2^ tests). This suggests that neurons classified in different groups show different response patterns. In addition, the interactions between these neurons also show different tendencies according to epoch.

### Enhanced decoding capability for behavioral responses due to neural interactions

**Fig. 5:**
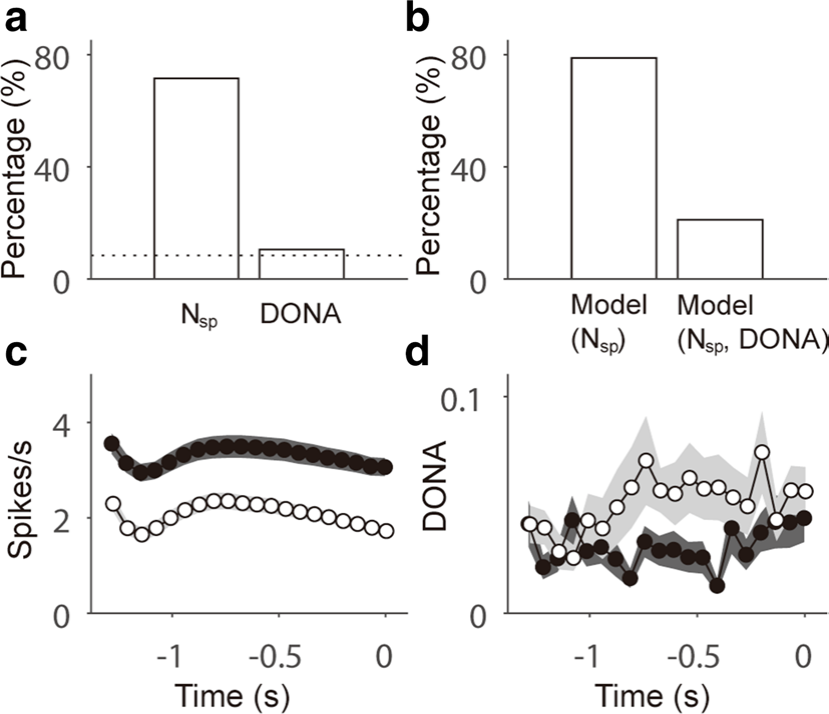
Analysis of optimal models via logistic regressions including DONA and spike counts ${N}_{sp}$. **a** Percentage of neurons for which spike counts or DONAs were significantly associated with the probability of the lick response (p < 0.05 in the logistic regression). The dotted line represents the significance level (binomial test, p < 0.05). **b** Percentage of neurons for which the optimal model (smaller AIC) in the logistic regression was the model including only spike counts and the full model including spike counts and DONA. **c**, **d** Example dynamics of the spike counts and DONAs of a neuron for which the probability of the lick response depends significantly on both variables of the neuron in the delay epoch. Solid and open circles represent the mean values of left and right lick responses, respectively, whereas shaded regions denote the standard error of the mean (SEM)

We performed logistic regression analyses with the spike count ${N}_{sp}$ and DONA to test whether neural interactions enhanced the performance when decoding lick responses (Fig. 5a; see Material and methods). The probability of the response was significantly dependent (p < 0.05 in the logistic regression) on DONA in 10.53% of neurons (p < 0.05, binomial test) and on the spike count in 71.58% of neurons (p < 0.01, binomial test), as shown in Fig. 5a. We compared the full model including both DONA and spike counts, which we expected to be optimal for single neurons, with the reduced model including only spike counts. Comparing the AICs of the two models, we found that the full model was optimal in a considerable number of neurons (21.13%; Fig. 5b). Thus, it seems that DONA can convey more information than spike count alone. In Fig. 5c, d, we compare the dynamics of DONA and spike counts according to lick responses by showing a representative example of a single neuron for which the probability of the response depended significantly on both DONA and the spike counts of the neuron in the delay epoch.

### Disparate trends of cross-correlation compared with DONA

**Fig. 6:**
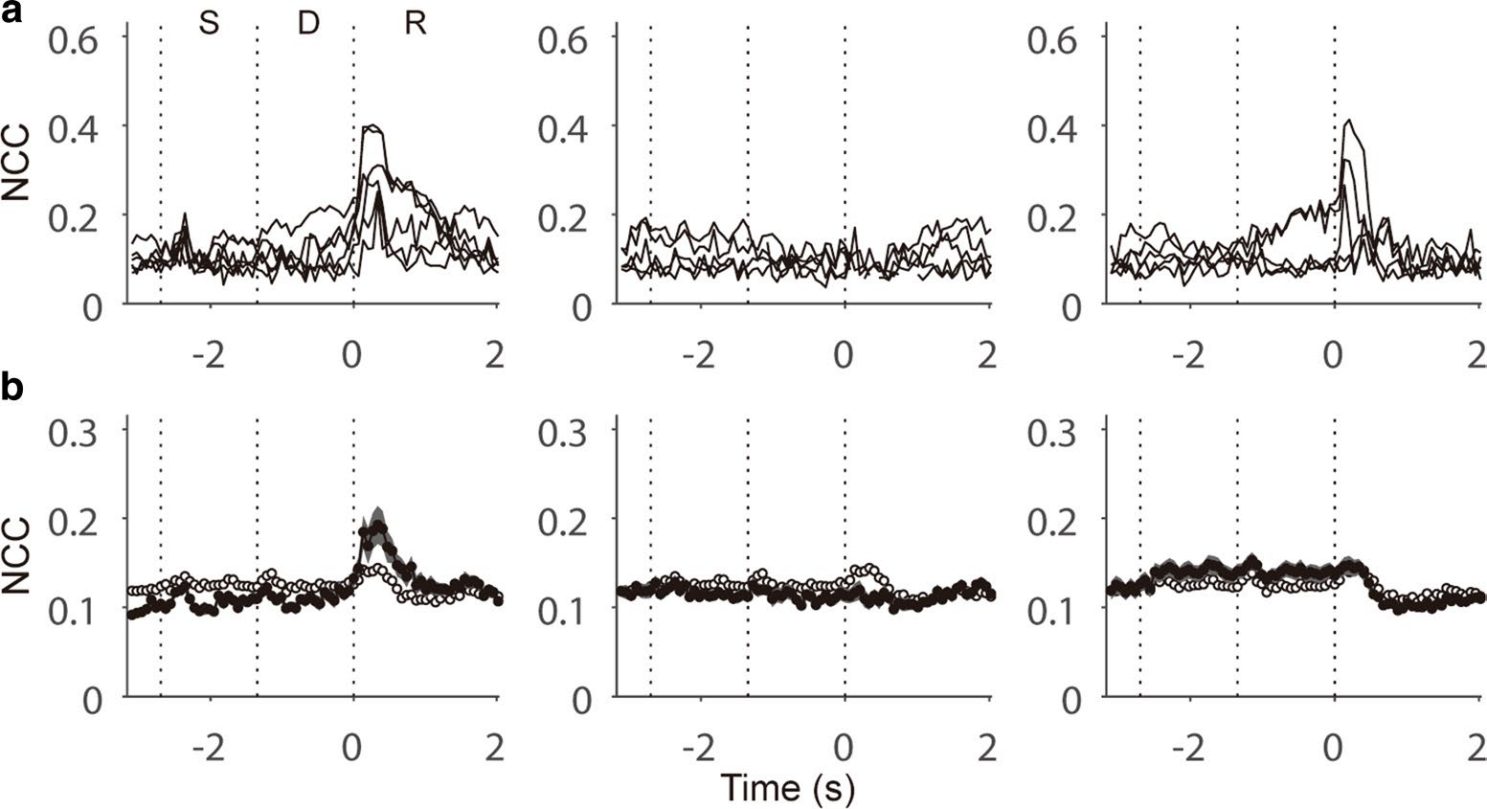
Normalized correlation coefficients (NCCs) for neurons sorted in the same manner shown in Fig. 3a, b. Open circles in (**b**) denote the mean NCCs of all neurons (the same format used in Fig. 3c)

To compare the localization trends of DONA (shown in Fig. 3a–c) with an existing and typical measure of pairwise interactions, we calculated NCCs for each neuron (Fig. 6; see Materials and methods). To enable the direct comparison of these measures, the results of NCC were arranged for the neurons sorted in the same manner shown in Fig. 3a–c. If NCC values could extract similar features of network topology as DONA, we would see a resemblance between NCC and DONA values in terms of localization to epochs. However, NCCs did not show such a localization tendency and the mean NCC showed no patterns along the epochs, unlike the mean DONA (Fig. 6b, open circle). These results suggest that DONA might extract nonoverlapping features related to the neural interactions in the population that cannot be extracted by the typically used correlation measure.

## Discussion

In the present study, we present a method by which to extract single-trial neural interactions using rate estimates obtained by a latent dynamical model. We applied this method to a dataset containing data from mice cortical neurons [22, 39–43]. The model applied in this method was able to evaluate the proportions of shared neural activities and transient effects evoked by other neurons; thus, it showed that inferred measures could trace the variation of neural interactions along epochs. Most neurons were maximally affected by other neurons in a specific epoch and, on average, neural interactions increased as an epoch progressed. To demonstrate the novelty of our method, we compared its performance to that achieved using cross-correlation coefficients; we showed that our model could reveal hidden features of the neural network that were not accounted for by a typical method in which pairwise interactions are estimated.

Obstacles must be overcome to ensure that our method can be generally applied to neural population data in different regions of the brain when performing diverse tasks. First, a number of existing models [15–29] have been established by which to analyze single-trial neural population dynamics and provide different rate estimates to certain extents. It might also be more challenging to estimate neural activities in single trials for tasks that require higher computational capabilities [21], although many studies have shown that population activities could be reduced to low-dimensional shared spaces [25, 30–37]. In such tasks, latent dynamical models provide suboptimal rate estimates and the consequential inference of neural interactions will mislead the coordination of the neural network. Hence, it will be necessary to analyze how discrepancies in rate estimates change the properties of DONAs or PDONAs and how the suitability of DONAs or PDONAs can be judged when using diverse datasets with varying levels of difficulty.

Second, timescales describing SNA and TSNA will vary according to types of neuron or task [22, 38]. Furthermore, correlations between neurons are identified in accordance with the timescale of their pairwise effects [12]. Consequently, it is vital that the temporal precision of the rate estimates and the transient effects are chosen appropriately to allow confident interpretation of neural interactions. In the present study, we chose the temporal precisions heuristically so that the analysis model showed results that were more distinctive. Therefore, we cannot exclude the possibility that models with different temporal precisions might provide new information about neural interactions. Additionally, for real-time processing, such as in brain–machine interfaces or closed-loop brain stimulations, temporal precisions must be automatically regulated depending on the given neural data. Thus, for the general application of our method, it will be essential to identify techniques and criteria by which to define temporal precisions according to the characteristics of the neural data.

Third, we only compared our methods to cross-correlation coefficients. A number of other measures exist for quantifying interactions that have various uses related to the characteristics of neural data or the purposes of analysis [10–14]. To validate the effectiveness of DONA and PDONA as measures for inferring neural interaction, comparisons to other measures, including the degree of synchronous firing or the temporal correlation of neural pairs, will be required to identify the similarities and differences. Indeed, we should be open to the possibility that combinations of single-trial rate estimates and measures of correlations might provide more information about network structure.

We anticipate that this study will be extensible for further research into neural population dynamics. Our developed method can provide novel insights into types of neurons or neural pairs based on neural interactions, just as neurons are classified into certain types based on their functional roles or responses in specific epochs or sessions [43]. Here we applied our method only to spike data with binary values. For its application to continuous datasets, such as those containing EEG or fMRI data, further research will be required. In particular, we must be able to define the shared activities and transient effects of continuous datasets in line with the measures applied in this work.

## Data Availability

The datasets supporting the conclusions of this article is available in the reference [42]. Interim data processed for supporting the conclusions of this article will be made available from the authors on reasonable request.

## Abbreviations

ALM: Anterior lateral motor
SNA: Shared neural activity
EDLDS: Event-dependent linear dynamical systems
CCG: Cross-correlogram
ECCG: Estimated CCG
DCCG: Difference between CCG and ECCG
TSNA: Transient SNA
ONA: Optimized neural activity
DONA: Kullback–Leibler divergence between ONA and SNA
PDONA: Pairwise DONA
NCC: Normalized cross-correlation
